# Ketogenic diets therapy in the management of epileptic spasms syndrome

**DOI:** 10.3389/fped.2024.1472982

**Published:** 2024-11-06

**Authors:** Meng Wang, Fen Zhao, Lina Sun, Yanyan Yu, Hongwei Zhang

**Affiliations:** ^1^ Epilepsy Center, Children’s Hospital Affiliated to Shandong University, Jinan, China; ^2^ Epilepsy Center, Jinan Children’s Hospital, Jinan, China; ^3^ Department of Special Function Examination, Anqiu People’s Hospital, Weifang, China

**Keywords:** ketogenic diet therapy, Infantile Epileptic Spasm Syndrome (IESS), refractory epilepsy, mechanisms, treatment

## Abstract

Infantile Epileptic Spasm Syndrome (IESS) is a group of infantile spasm syndromes of various etiologies that typically present in early infancy, predispose to refractory epilepsy, and leave intellectual disability. Ketogenic diet therapy (KDT) is a non-pharmacologic treatment modality for medically refractory IESS. Recent scientific evidence supported the efficacy, safety, and tolerability of KDT for the treatment of IESS. KDT not only reduces the frequency of seizures in infants with IESS, but also improve their cognition and long-term prognosis. Recently, it has also received increasing attention as a potential treatment for neurological disorders. This reviewed the recent research progress of KDTs for the treatment of IESS, and discussed the different types and the mechanisms of KDTs, the expansion of KDT applications, the influencing factors, and future research issues.

## 1 Introduction

Infantile Epileptic Spasm Syndrome (IESS) is an age-specific epilepsy syndrome that occurs in early infancy (1), characterized by epileptic spasms, hypsarrhythmia on electroencephalogram (EEG) or developmental regression, which must not inevitably exist before the onset of spasms (2, 3). There is an incidence rate of 2–6 per 10,000 live births with a prevalence of 1–2 per 10,000 children under the age of 10 (1). The etiology of IESS is heterogeneous, with approximately one third of cases having no known etiology (1, 4). In the pathogenesis of IESS, factors such as synaptic abnormalities, nerve growth factors, hypothalamic-pituitary-adrenal axis, and inflammation might play an important role in the development of IESS (5). However, current researches about the pathogenesis of IESS is based on animal studies and involves fewer human studies.

In the treatment of IESS, there is no universally accepted mainstay of treatment for this condition. American Academy of Neurology indicated that low-dose adrenocorticotropic hormone (ACTH) was the first-line pharmacologic therapy for IESS regardless of etiology (6). In Japanese, the drug of choice varied based on etiology and included synthetic ACTH, pyridoxine, and valproate (7). In UK, it was found that vigabatrin was the most common first-line agent (7). Surgical treatment was only appropriate for those with definite epileptogenic focus. And deep brain stimulation has limited seizure-free rate for those unresponsive with antiseizure medications (ASMs) (7). However, despite the above proper treatments, a quite number of patients remain drug-resistant epilepsy and progressive intellectual disability (8). Therefore, new therapies are urgently needed to broaden the management options and improve the prognosis of those with IESS.

The KDT is a very low-carbohydrate, high-fat, and adequate protein nutritional approach that induces a metabolic shift to the use of ketone bodies as an additional energy source (8). It was initially introduced as a treatment for epilepsy. However, the introduction and development of ASMs in 1938, declined the use of the KDT almost completely (8). Since the mid-1990s, KDT has gained attention again as an alternative treatment for refractory epilepsy in children due to its improvement of cognition. The use of KDT is reversible, inexpensive, and readily available compared to surgical treatment. At present, it is recognized that good effectiveness and safety of KDT in the treatment for drug-resistant epilepsy (9). However, data of clinical studies of a KDT as a treatment for IESS are limited and lacked a systematic evaluation. Therefore, the present review examined the role of a KDT in IESS treatment and discussed the underlying mechanisms, aims to present novel perspectives for the development and implementation of IESS.

## 2 The classification of KDT

KDT is now categorized into the classic ketogenic diet (cKD), a medium-chain triglyceride (MCT) diet, a modified Atkins diet (MAD), and a low glycemic index treatment (LGIT). They share the common characteristics of high fat, low carbohydrates, and moderate protein, while differing in the ratio of bulk nutrients and the ketogenic ratio.

The classic KDT was most prescribed in children, accounting for 60%, followed by MAD (25%) and MCT (10%). The LGIT is prescribed in around 5% of epileptic patients (8). The lipid-to-nonlipid (total protein and carbohydrate) weight ratio, known as the ketogenic proportion, is usually determined as 4:1, 3:1, or 2:1. In classic KDT, the ketogenic proportion can range from 1:1 to 4:1, depending on individual therapeutic needs. The most desired proportion in clinical conditions is 4:1, providing 80% of the total energy from fat, primarily long-chain triglycerides. The higher the proportion, the more restrictive and theoretically more effective the diet is. In children with IESS, the ketogenic proportion may be reduced to 3.5:1 or 3:1 due to the need for protein for growth and development, which allows for higher carbohydrate intake and improves the acceptability and tolerance of classic KDT (10). The MCT diet, with medium-chain triglycerides as the main source of fat (11). It is more lenient compared to the classic KDT, with a fat intake of about 70% of total calories. MCT can be absorbed and transported directly through the portal vein to the liver to produce ketone bodies. This property allows MCT to produce more ketones, and consume more carbohydrates and protein and less fat. Medium-chain triglycerides produce more ketones per calorie of energy than long-chain triglycerides, and the increased ketogenic potential of the MCT diet allows for reduced total fat intake and increased carbohydrate intake, which enriches the diet (12). The MAD could restrict carbohydrate intake, with a ketogenic proportion of 1:1–1.2:1. The initial daily carbohydrate restriction for MCD is approximately 20 g/day with no restriction on protein or caloric intake, and the meal plan is more extensive. Thus, the MAD is easy to operate and perform, and patient compliance is higher than classical KDT. It can usually be started on an outpatient basis due to no fasting period (13). The LGIT was created for stabilizing glucose levels in KDT (14). In LGIT, total daily carbohydrate intake is about 40–160 g/day to keep blood glucose levels stable in the brain. All carbohydrates are glycemic index below 50. The protein and fat intakes are also monitored, but not as strictly as in the classical KDT (15).

All KDTs were suitable for the treatment of drug-resistant epilepsy. Other than epilepsy, these KDT protocols are being explored more and more as possible treatments for a variety of conditions, like autism spectrum disorders, endocrine disorders, and Alzheimer's disease. However, it is acknowledged that all KDTs have a certain percentage of attrition rate due to adverse effects and a lack of effectiveness. In infants with IESS, the attrition rate is lower due to the easier control of the diet by their caregivers. Patients with drug-resistant seizures need long-term dietary therapy continued for approximately 2 years (8). Therefore, the management of KDT is a long-term program that requires regular nutritional testing to ensure its effectiveness and avoid malnutrition or overnutrition (16).

## 3 KDT mechanisms in the treatment of IESS

At present, the real mechanisms of the KDT's anti-seizure effects still remain unclear, although many potential mechanisms have been discovered. Recent studies have indicated that the liver and astrocytes were the sites where the polyunsaturated fatty acids are broken down to produce ketone bodies (17, 18). Polyunsaturated fatty acids are oxidized in the mitochondria to produce energy, and at the same time produce acetyl coenzyme A(Acetyl-CoA). A large amount of Acetyl-CoA produces acetoacetic acid and β-hydroxybutyric acid in the liver or under the astrocytes, and later enter the bloodstream to produce acetone. Ketone bodies can replace glucose as the main energy source of the brain, where ketone bodies are converted to Acetyl-CoA, which is metabolized by the mitochondria to produce adenosine triphosphate (ATP) (19).

**Figure 1:**
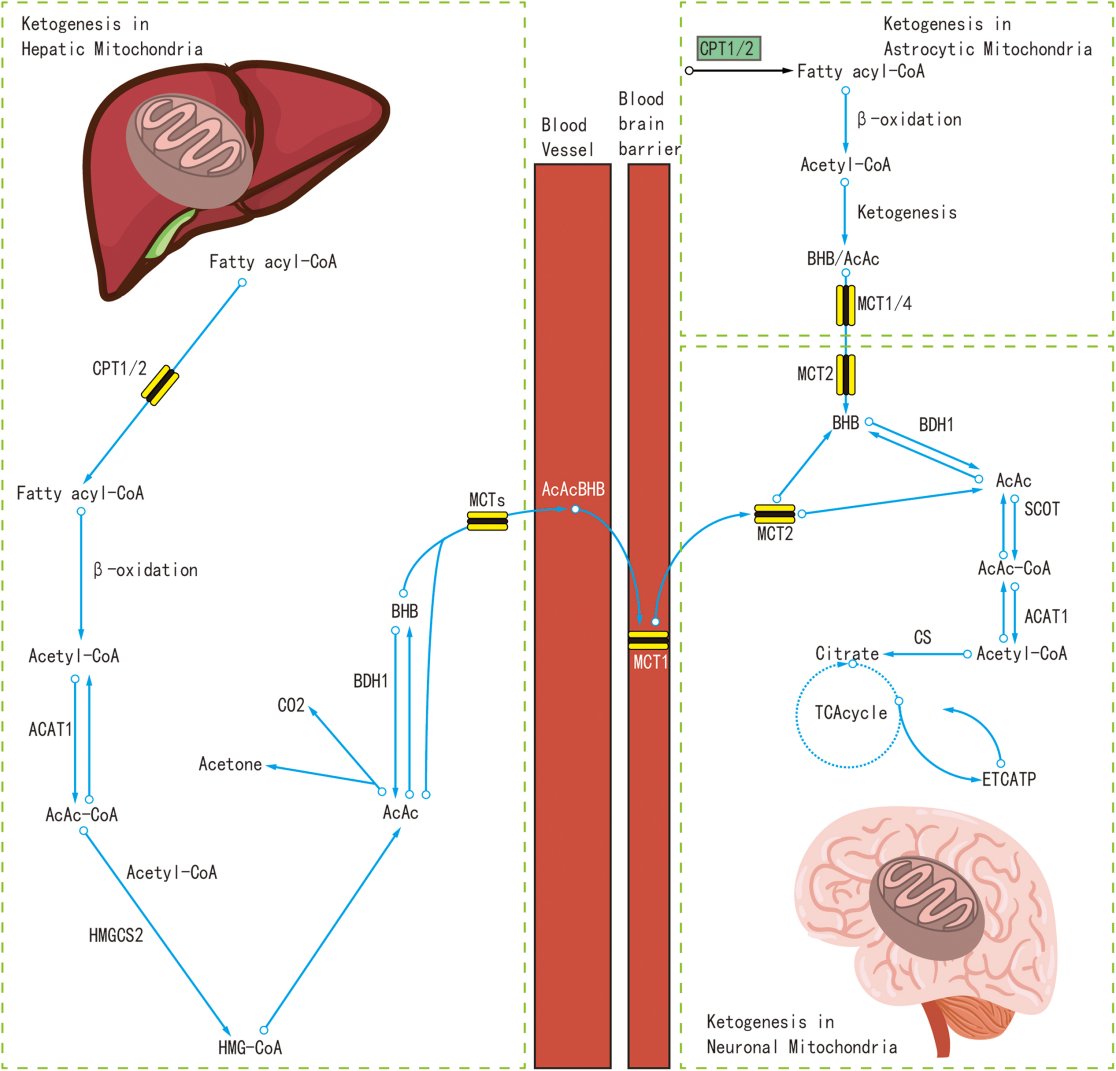
The mechanism of KDT.

The mechanism of KDTs in the treatment of IESS was not a single, but multiple mechanisms likely participate in interconnected ways to produce anti-seizure effects (18). These mechanisms probably act jointly and in parallel with each other. In the following, we will mainly discuss several potential mechanisms of KDT in the treatment of IESS, as shown in Figure 1.

### 3.1 Effect of KDT on neurotransmitters

Children with IESS may have an imbalance between inhibitory and excitatory neurotransmitters, which can lead to seizures and neurotransmitter abnormalities that further impair cognitive function. The antiepileptic mechanism of KDT is to modulate the balance between these neurotransmitter systems, which further improves cognitive function.

First, KDT attenuates the effects of excitatory neurotransmitters, such as monoamine neurotransmitters and glutamate (18, 19). Monoamine neurotransmitters, including norepinephrine, serotonin, and dopamine, play an important role in controlling neuronal excitability and seizures. Previous animal studies have shown that KDT has no anticonvulsant activity in norepinephrine-deprived animals (19), KDT also increases norepinephrine in the extracellular fluid of the hippocampus (20), and clinical studies have shown that KDT can affect serotonin and dopamine levels in the cerebrospinal fluid (CSF) of children with drug-resistant epilepsy (9). Glutamate is an important excitatory neurotransmitter in the brain that can make the brain susceptible to seizures (19), and the effect of KDT on brain glutamate metabolism is still controversial (21–23), and further studies are needed.

Secondly, KDT has been demonstrated to enhance the effects of inhibitory neurotransmitters, including gamma-aminobutyric acid (GABA) and agmatine. Gamma-aminobutyric acid (GABA) is capable of inhibiting neural networks through the action of GABAA receptors (24, 25). In the immature brain, GABA plays a significant role in neuronal value-adding, migration, and the formation of neural networks (26). In KDT, it also It is also important in neuronal value-adding, migration, and neural networks (26, 27) In KDT, the conversion of glutamate to GABA in neurons is increased while the degradation of GABA is decreased (27), which further increases the amount of GABA.

### 3.2 Effect of KDT on ion channels

In KDT, various pathways can be used to open KATP channels, leading to neuronal membrane hyperpolarization and increased seizure thresholds (28–31), resulting in fewer seizures (32). Other studies have shown that acetoacetate suppresses seizures *in vivo* by inhibiting voltage-dependent Ca2+ channels (VDCCs) and decreasing excitatory postsynaptic currents (EPSCs) at sites of epileptic activity (31).

### 3.3 Effect of KDT on growth factor

Previous studies have shown that cerebrospinal fluid insulin-like growth factor-1 (IGF-1) concentrations are significantly lower in children with IESS of known etiology (33), and that cerebrospinal fluid IGF-1 levels have been associated with early stress, cortical damage, poor response to treatment, and poor cognitive outcomes (5), and that it may also lead to abnormalities in neuronal development and synapse formation, which may affect disease severity and prognosis (33). The KDT may result in a reduction of insulin-like growth factor-1 (IGF-1) levels and may also influence the activity of other growth factors by affecting the insulin-like growth factor signaling pathway. However, more research is needed to confirm the specific effects of the ketogenic diet on growth factors.

### 3.4 Protective effects of KDT on neurons

#### 3.4.1 KDT reduces inflammatory response

Currently, the role of inflammation in IESS can be demonstrated indirectly, for example, by examining cellular levels in blood and cerebrospinal fluid (3) or by indirectly detecting underlying inflammation through the selective efficacy of ACTH in IESS (34, 35). The anti-inflammatory capacity of the KDT is multifaceted, modulating both central and peripheral inflammatory mechanisms (36). This occurs by reducing microglia activation and proinflammatory factors in the hippocampus, and by suppressing neuroinflammation through the inhibition of, for example, cyclooxygenase 2 (COX-2) (37–40).

#### 3.4.2 KDT modifies energy metabolism and oxidative stress

In IESS, oxidative stress may be associated with a variety of factors, including brain injury, inflammation, and neurotransmitter imbalances (5). KDT modulates uncoupling proteins. This results in a reduction in ROS production, protection of neurons from oxidative stress, and enhancement of seizure resistance (41). Additionally, KDT protects against seizure damage by increasing glutathione levels (42). Furthermore, KDT treatment has been observed to increase polyunsaturated fatty acids, which have been demonstrated to induce the expression of neuronal uncoupling proteins, regulate numerous genes involved in energy metabolism, and induce mitochondrial biosynthesis. This ultimately results in a reduction in ROS production and an increase in energy (43–45).

#### 3.4.3 mTOR pathway

Disorders of the mammalian target of the rapamycin (mTOR) pathway are associated with IESS and dysplastic tissue caused by mTOR activation is associated with IESS (46). Excessive mTOR activation results in abnormal cell proliferation, tumor formation, and dysplastic cells, leading to tuberous sclerosis complex (TSC). Up to 25% of patients with IESS are diagnosed with TSC (47, 48). There may be a mechanistic link between KDT and mTOR, acting through amino acid metabolism on mTOR signaling (49, 50).

#### 3.4.4 KDT reduces nerve cell death

Neuronal injury and death lead to neuronal deficits and abnormal brain function, which is associated with the development of IESS (5). Additionally, the extent of neuronal damage caused by seizures may exacerbate the cognitive deficits and the severity of seizures in patients with IESS (51, 52). KDTs have been shown to reduce neuronal damage by inhibiting pro-apoptotic factors such as cysteine and other pro-apoptotic factors. Prior research has indicated that KDTs may mitigate the deleterious effects of these processes (53). KDTs inhibit pro-apoptotic factors, such as cysteine, thereby reducing neurological damage. Furthermore, KDTs act as intracellular calcium buffers by upregulating calmodulin, thereby protecting nerves (54). The process of neuronal death is influenced by a multitude of factors, including autophagy, phagocytosis, necrosis, and apoptosis. Further investigation is required to elucidate the effects and mechanisms of KDTs on these factors.

### 3.5 Effects of KDT on gut microbiota

It has recently been demonstrated that KDT can exert an influence on seizures through the action of gut microbes (GM). This influence may be exerted by the production of neurotransmitters (55) or neuropeptides, or by affecting the expression of GABAH and n-NMDA receptors in the brain (56). Prior animal studies have demonstrated that KDT can mitigate acute seizures by modulating the gut microbiota (57), a finding that has been corroborated in clinical investigations (58). Currently, there is a degree of debate surrounding the hypothesis that KDT exerts an influence on epilepsy by modulating the number of *bifidobacteria* (59–61). Further research is required to substantiate this proposition. Additionally, evidence from some studies indicates that GM can influence seizure frequency and seizure threshold by affecting inflammatory mediators.

## 4 KDT for IESS

### 4.1 KDT for IESS clinical outcomes

The efficacy of the ketogenic diet in the treatment of IESS has been substantiated in clinical trials. Recent studies have shown >50% spasticity remission rates of 75%, 82.6%, and 90.9% after 3, 6, and 12 months of classic KDT, respectively (62). The results of this study are similar to those of many previous studies (63). A systematic review of the treatment of WS conducted by G. Prezioso et al. included 13 studies with a total of approximately 300 patients. The results of the study showed that in the short term, the KDT reduced seizures by more than 50% in about 60% of the patients, and about 35% of patients were completely seizure-free. However, due to limited data, the long term effects are currently unknown (64). The results of another study on the role of KDT in childhood epilepsy, conducted through a review and meta-analysis of randomized controlled trials, demonstrated that the KDT group exhibited a 5.6 times greater likelihood of experiencing a 50% reduction in seizures after a three-month dietary intervention, or at an earlier point in time. The study included a total of 453 patients. The study population was selected as follows: 184 patients were given the MAD diet, 54 were kept on the classic 4:1 ketogenic liquid formula, and the remaining 215 patients were treated with the standard therapy (ST). This article was quality assessed by QUADAS and AMSTAR, which demonstrated a low risk of bias and adequate accuracy (65).

Previous studies have demonstrated the potential of the ketogenic diet to alleviate seizures in patients with IESS. In some cases, patients with IESS have achieved complete seizure remission following the implementation of a ketogenic diet regimen (62–64). Not only does the ketogenic diet result in a reduction in seizures, but studies have also indicated that children patients with drug-resistant epilepsy who adhere to the ketogenic diet may experience a discontinuation of or a reduction in the necessity for antiepileptic drugs (66). The current research on KDT in the treatment of IESS has focused on four main areas: the effect of the ketogenic diet as a first-line treatment, the effect of the ketogenic diet as a second-line treatment, the effect of different ketogenic diets, and other effects of the ketogenic diet on IESS.

There is some controversy about the effectiveness of KDT as a first line of treatment. The results of a recent prospective PC-RCT in IESS showed that the overall efficacy of the two treatments, KDT and ACTH, was similar (electroclinical remission rate at day 28 KDT: 27%, ACTH: 48%), but KDT was better tolerated (63). This finding is similar to the results of a previous retrospective study of infants with new-onset IESS comparing 13 infants treated with KDT to 20 infants treated with ACTH, In this retrospective study, the KDT was observed to have a nearly two-thirds success rate in stopping spasms, with fewer adverse effects and relapse rates than ACTH. However, ACTH normalized the EEG more rapidly (62). However, A national multicenter retrospective study of IESS complicated by Leigh syndrome (LS) and Leigh-Like Syndrome conducted in 2024 revealed that four of nine patients (44%) treated with ACTH achieved clinical electrical remission within one month of treatment. Additionally, one of seven patients (14%) treated with KDT achieved clinical electrical remission within the same time frame. It is noteworthy that none of the patients treated with antiseizure medications (ASMs) only achieved clinical remission. However, a national multicenter retrospective study of IESS complicated by Leigh syndrome (LS) and Leigh-Like Syndrome(LLS) conducted in 2024 revealed that four of nine patients (44%) treated with ACTH achieved clinical electrical remission within one month of treatment. Additionally, one of seven patients (14%) treated with KDT achieved clinical electrical remission within the same time frame. It is noteworthy that none of the patients treated with antiseizure medications (ASMs) only achieved clinical remission. The relatively small sample size of 21 patients in this study may limit the generalizability and statistical significance of the findings. Furthermore, due to the retrospective nature of the study, there is a possibility of bias and incompleteness in the data collection process (67). The preceding discussion demonstrates that the therapeutic efficacy of KDT in comparison to first-line drugs is a topic of contention and necessitates the inclusion of larger sample sizes and a more comprehensive range of research methodologies.

Although current studies indicate that KDT exhibits comparable therapeutic efficacy to first-line medications, the limited sample sizes of KDT as a first-line treatment make it challenging to conduct higher-level studies.

More studies have shown good efficacy of KDT as a second line treatment. The KDT is now generally used for refractory IESS after initial drug therapy has failed (68). Previous studies of 104 patients with refractory IESS treated with KDT showed that approximately 18%–33% of patients were completely free of spasticity for 3–24 months, and approximately two-thirds of patientshad a greater than 50% reduction in spasticity after 6 months of ketogenic treatment. The results suggest that KDT should be a strong consideration for IESS after the failure of corticosteroids and vigabatrin therapy (69). A study of 39 patients with medically refractory IESS showed that 61.5% of patients had a greater than 50% reduction in seizures within 6 months of KDT treatment, and 4 of them were completely seizure-free (70). The results of the above studies show that KDT is an effective treatment for IESS.

There is some controversy about the effect of different types of KDTs on the therapeutic efficacy of IESS. A recent open-label, randomized, controlled trial in patients aged 9 months to 3 years with first-line refractory epileptic spasms showed that MAD and CKD were comparable in the proportion of patientswith epileptic spasms who achieved spasm relief (71). This is similar to some previous studies (72–74). However, this result is somewhat controversial, as some studies suggest that the treatment response rate is higher with CKD, and in previous randomized experimental studies in 1–2-year-old patients, the seizure rate was higher in patients with CKD than in patients with MAD (53% for CKD, 20% for MAD) (72). The reason for this difference is currently unknown and may be related to individual differences, type, and severity of disease. In addition to study in Chinese pediatric patients with epileptic spasms found that MAD is more effective in controlling spasticity seizures on a long-term basis (75). Another study have also shown that MAD is better tolerated and more easily accepted and adhered to by patients and families (76).

Moreover, KDT has been shown to facilitate improvements in electroencephalogram (EEG) abnormalities in the IESS (70). For IESS caused by mutations in specific genes, KDT has been shown to be an effective therapy. Some previous case reports have shown good results in treating infants with genetic mutations in ALG13 (77), SCN2A (78), MEDS (79), and CDKL5 (76). A prior study indicated that KDT may reduce stress levels in families of children with refractory epilepsy. However, the study was conducted at a single center with a limited sample size, and further research is necessary to substantiate these findings (80).

Due to the variable efficacy of KDT in individual patients, research has been conducted to identify the factors that influence the efficacy of KDT therapy. These factors include etiology (64), age (69), gender, medications, KDT initiation time (81), blood ketone level, etc., but the effect of their influence is still controversial and needs further research (70).

In conclusion, the therapeutic efficacy of KDT in the treatment of IESS is evident. However, it is also important to acknowledge the potential adverse effects of KDT in IESS treatment. Previous studies have demonstrated that the primary adverse effects associated with the ketogenic diet are vomiting and constipation, and other side effects such as somnolence, weight loss, dyslipidemia, metabolic acidosis, and kidney stones. There are some reports of growth retardation in patientstaking KDT for long periods (82), but this is controversial and should be studied further. Prebiotic supplementation has been suggested in animal models to reverse the effects of KDT on blood metabolism and reduce the side effects caused by KDT (83).

### 4.2 The predictive factors of the effectiveness of KDT in the treatment of IESS

Prior research has demonstrated that improvement in EEG findings early after CKD treatment may help predict children's response to treatment (70). Kramer's method for quantifying the severity of hypsarrhythmia on the EEGs is now in use (84). The score of hypsarrhythmia on the EEGs after 3 months of CKD treatment is associated with various epilepsy outcomes. Adverse epileptic outcomes can be predicted by the quantitative cutoff of the hypsarrhythmia score criteria (calculated as ≥8), which has high sensitivity and specificity. Therefore, patients with no clinical improvement after KDT or a hypsarrhythmia score ≥8 are predicted to have a poor long-term outcome, and alternative treatments should be recommended (62).

## 5 Conclusions

According to the randomized clinical trials presented in the review, KDT is effective in treating IESS. Still, the limited number of trials and the small number of patients resulted in a poor overall quality of the studies, and further research is needed. KDT can be used in different populations of IESS and is a safe treatment option. Several less restrictive KDTs, including MCT, MAD, and LGIT, can be considered in the treatment process due to their better tolerability, lower treatment costs, ease of administration, and greater acceptability. However, their therapeutic efficacy still needs to be further validated in clinical trials.

## 6 Future directions

In previous studies of KDT for IESS, the sample size of patients in many studies was small. Therefore, more large randomized controlled trials are needed to validate the issue of KDT efficacy. Furthermore, there is a paucity of research examining the enhancement of children's quality of life, patients’ cognition, growth problems, and the impact on the patient's family (e.g., cost, psychology, social relations, etc.). The last but not least, the mechanisms and special biomarkers in the process of the study of IESS require further investigation to establish a biological foundation for clinical treatment.
